# Child Abuse and Neglect: Awareness among Dental Students

**DOI:** 10.3390/healthcare11182510

**Published:** 2023-09-11

**Authors:** Manal Almutairi, Ibrahim Alomran, Reema Alshahrani, Rehaf Alsania, Hafsah Al Ali, Rehab Allam, Noura Alessa, Zain Hafiz

**Affiliations:** 1 Department of Pediatric Dentistry and Orthodontics, College of Dentistry, King Saud University, Riyadh 11545, Saudi Arabia; rallam@ksu.edu.sa (R.A.); nalessa@ksu.edu.sa (N.A.); zhafiz1@ksu.edu.sa (Z.H.); 2 College of Dentistry, King Saud University, Riyadh 11545, Saudi Arabia; 437104211@student.ksu.edu.sa (I.A.); 439200321@student.ksu.edu.sa (R.A.); 439200513@student.ksu.edu.sa (R.A.); 437201855@student.ksu.edu.sa (H.A.A.)

**Keywords:** attitudes, awareness, child abuse, dentistry, students

## Abstract

Child abuse and neglect (CAN) have significant consequences for children’s health and well-being. Dentists play an important role in identifying and reporting CAN cases. This study aimed to assess knowledge and attitudes toward CAN among dental students. A cross-sectional study was conducted among dental students, comparing second-year (preclinical) and fifth-year (clinical) students, both male and female, in the College of Dentistry at King Saud University during the first semester of the 2022/2023 academic year, using a questionnaire with 23 multiple-choice questions and an electronic link. The results revealed that most dental students (96%) were aware of CAN, with social media being the most utilized source of information (85%). However, the dental college was noticed as the least utilized source (50%). Most dental students recognize the significance of CAN in dentistry and expressed an intense interest in learning how to effectively deal with such cases. Only a small proportion, 16%, of dental students reported experiencing incidents of CAN in their clinical practice. In general, there were statistically significant differences between preclinical and clinical students, but there were no significant differences in awareness between female and male students when the source of information and role in dealing with CAN were taken into consideration. Both preclinical and clinical dental students were aware of CAN, although there were concerns about their ability to cope with it. The results emphasize the importance of comprehensive education and training programs across the dental curriculum about CAN.

## 1. Introduction

Child abuse and neglect (CAN) constitute a significant problem in the lives of children from all over the world, and it influences them in several different areas of their lives [1]. Physical abuse, mental abuse, sexual abuse, or neglect are all types of injury that are classified as CAN for children under the age of eighteen [1]. It can result from a combination of individual child factors, parental factors, social, and/or environmental risk factors without protective factors [2].

The World Health Organization (WHO) estimates three out of every four children experience physical and/or mental abuse, which highlights the pervasive incidence of CAN worldwide [3]. As a result, a broad range of long-term health problems has been associated with CAN, including depression, anxiety, drug use, suicide attempts, and sexually transmitted infections [4]. In the United States, the most frequent form of child abuse is neglect, which is compatible with other forms of abuse and accounts for 66.7% of all cases of CAN and 1750 children annually [5].

According to a recent meta-analysis [6], the worldwide prevalence rates for CAN vary from 0.3% for studies using professional reports to 36.6% for self-report studies. Furthermore, the prevalence of adults who have experienced childhood sexual abuse has been demonstrated in the literature to be 1:13 for men and 1:5 for women [2]. A study conducted in Saudi Arabia, ref. [7] revealed that psychological abuse makes up 74.9% of all abuses, with physical abuse followed at 57.5%, neglect at 50.2%, and sexual abuse at 14.0%. According to a systematic review by Nilchian et al., a significant portion of child abuse is unreported every year for a variety of reasons, such as failure to gather a history, diagnostic uncertainty, and concern about familial violence against children [8]. In conjunction with the occurrence of child physical abuse and the insufficiency of official statistics reliant on complaints made to social services, law enforcement, and medical institutions, nations have increasingly turned to data originating from the educational and healthcare domains [9]. In 2009, the Ministry of Social Affairs in Saudi Arabia issued a directive requiring the education and medical domains to promptly inform the police of any potential child abuse instances, as they are the authorized entities to receive and manage such reports. Suspected abuse cases will then be forwarded by the police to a specialized committee comprising professionals from various fields, including a pediatrician, pediatric surgeon, pediatric dentist, psychiatrist, and social worker. This expert team will work collaboratively to conduct a thorough investigation into the case [10].

Dentists/forensic odontologists have a distinctive chance to observe and interact with children, placing them in a good position to detect signs of abuse in the craniofacial and oral regions like slap marks, squeezed ears, or bite marks, and to provide support [11]. Since punishment by corporal force is frequently used in our society, doctors and dentists require criteria for when it is excessive and hence indicative of physical abuse [11].

To report their suspicions to the proper authorities and implement the required corrective measures to ease the suffering of the affected child, dentists must be aware of their moral, ethical, and legal responsibility regarding CAN. Additionally, they must receive in-depth instruction on CAN during their undergraduate and graduate degrees. It is also critical to promote understanding of the condition by providing in-service training at the facilities where they work [12]. In the study by Sulimany et al., it was found that only 39.5% of the 988 dental graduates who participated in the evaluation of knowledge levels and educational experiences for CAN felt confident in their capability to recognize a CAN in the case, and only 9.7% were aware of the procedures for reporting such incidents [13]. Additionally, a fraction of cases could be inadequately addressed due to underreporting by physicians, attributed to factors including, though not confined to, taking into account families’ statements and a lack of familiarity with referral protocols [14]. Another study reveals that almost half of physicians and students perceive non-life-threatening abuse as disciplinary actions and culturally acceptable practices [15]. According to a questionnaire-based study, 79% of Saudi Arabian professionals in public, private, and nonprofit organizations reported that CAN existed in the kingdom, and 42% were aware that Saudi Arabia had institutional organizations in place to protect children [16].

Similar studies to ours were performed at local Saudi Arabian universities, including one comparing interns with medical students [17,18]. It was determined that medical students had appropriate knowledge about CAN, but they did not know how to report CAN cases [17]. Professionals in healthcare, such as doctors, should receive training to identify, record, and prevent all CAN cases early on in their medical education, especially those that have severe and protracted negative effects on victims, such as child sexual abuse, so that the professionals can be trained, and the environment is ready to handle the expected victims with confidence [18]. 

To our knowledge, there have been limited studies evaluating undergraduate dental education in Saudi Arabia in terms of CAN. Therefore, the aim of our study is to assess dental students’ awareness by comparing preclinical and clinical males to females, assess their opinions on the necessity for supplementary education in the College of Dentistry at King Saud University in Riyadh, Kingdom of Saudi Arabia, and identify the most frequently accessed source of information for dental students about CAN.

## 2. Materials and Methods

The current observational study is being carried out in strict accordance with the Helsinki Declaration of the World Medical Association. The Institutional Review Board provided its ethical approval, as well as the Ethics Committee of the College of Dentistry Research Institute (IRB number E-22-7152). All participants agreed to provide informed consent.

A cross-sectional study was carried out to study CAN awareness and compare the preclinical year, defined as the second dental year, and clinical year, defined as the fifth and final dental year, of male and female dental students at King Saud University, Riyadh, Kingdom of Saudi Arabia, during the first semester in 2022/2023. Following the power calculation, the overall sample size for this study meant at least 66 dental students were needed at a significance level of 0.05, effect size of 0.75, and power of 0.85. 

All dental students, both male and female participants from the second (preclinical) and fifth (clinical) years, were invited to participate in this study. 

The data were collected from 75 student participants using an electronic survey, which was sent to the students by email, which was taken with permission from the college administration. The survey was standardized and completed via Google Forms, an online electronic form. The questionnaire was written in English and developed based on previous studies [19,20,21,22]. The questionnaire consisted of 23 multiple-choice questions designed to assess students’ knowledge about and abilities to detect child abuse. Simple descriptive statistics were used to assess each question. Two pediatric dentists evaluated the questionnaire’s content validity. They were asked to assess the accuracy, phrasing, and importance of each topic in the questionnaire. Minor changes to the questionnaire were made in response to expert feedback to improve the comprehensive nature of the questions. The face validity of the questionnaire was evaluated by distributing it to a group of dental students (n = 10) to evaluate its clarity. Furthermore, test–retest reliability was evaluated by having ten dental students complete the questionnaire twice, at one-week intervals. It also contained a cover letter aimed to survey the respondents’ characteristics and demographics. To maintain information confidentiality, all participant data were coded. Cluster random sampling was used, with the first sample (population forms of a second dental year) and the second sample (population forms of a final dental year) selected, and then all units in each of the selected clusters were surveyed at random. To enhance response rates, three reminders were issued to dental students at two-week intervals. Following that, questionnaires were collected from each level representative. To enhance the sample’s representativeness, only responses from those who answered were evaluated.

Data analysis was performed using the Statistical Package of Social Sciences (SPSS) version 26 (IBM Corp., Armonk, NY, USA). Descriptive statistics were used to obtain frequency and percentages. Finally, the chi-square test was used to discover whether significant differences existed between the participants in mean knowledge and attitude scores. Statistical significance was taken at *p* < 0.05.

## 3. Results

The study included 75 student participants. The demographic and background findings of the participants are presented in Table 1. There were 43 (57.3%) preclinical/second-year dental students and 32 (42.7%) clinical/fifth-year dental students. About 72 (96.0%) dental students were aware of CAN, and the most important source of information for 64 (85.3%) of participants was social media, while 38 (50.7%) of the participants indicated their awareness originated from their dental college education, which also appeared to be the least important source of information.

According to Table 2, approximately 70 (93.3%) of dental students believe that child abuse and neglect are essential topics in the dental field. Around 17 (23.0%) dental students have witnessed a case of child abuse and neglect throughout their time as students. In the Saudi population, 71 dental students (94.7%) believed in CAN, while 26 (34.7%) of the dental students believed that CAN is common in the Saudi community. Seventy-two (96.0%) of the dental students thought CAN would have influenced the child’s future. Half of the dental students believed that as future dentists, they will be involved in dealing with CAN in any discipline. Sixty-six (88.0%) of the dental students were interested to know the best way of dealing with CAN. Almost half of the dental students had taken academic courses or sessions on CAN at their dental college. The majority of those who had completed academic courses or sessions believed that the courses were fair, sufficient, or insufficient, and 53 (70.7%) of all dental students preferred to have more courses or sessions on CAN in the Dental College at King Saud University, Riyadh, Kingdom of Saudi Arabia.

Table 3 summarizes the opinions of dental students. Dental students were asked about their opinions on the legal systems that deal with CAN and preventive measures. Most dental students thought that social workers, physicians, or police dealt with the issue of CAN, as indicated by 60 (80.0%), 60 (80.0%), and 57 (76.0%) of the dental students, respectively. The dental students’ thoughts about whether there is a legal system—legislation—to deal with CAN in Saudi Arabia showed that 71 (94.7%) of them feel there is a legal system. A total of 31 (41.3%) dental students expected that psychological disorders in abusers were commonly involved, followed by always involved in 17 (22.7%) of dental students. Legalization and law enforcement were chosen by nearly half of the students as the most effective measures to raise awareness about CAN 35 (46.6%), followed by public education 28 (37%). When students were asked what they would do first if they encountered a case of child abuse in the clinic, only 33 (44%) stated they would send it to the police as a medico-legal case.

Table 4 shows the results of the comparison of genders and academic years with each characteristic. When asked who is responsible for children who have been abused or neglected, there was a statistically significant difference (*p*-value = 0.011) between female and male dental students. Most female dental students, 34 (91.9%), agreed that social workers are responsible for children who have been abused or neglected, whereas only 26 (68.0%) of male students agreed. When it came to opinions regarding dental college as a source of information about child abuse, there was a highly significant difference between second-year students (six; 14.0%) and fifth-year students (thirty-one; 96.9%) with a (*p*-value = 0.000), but no statistical significance between males (twenty; 52.6%) and females (seventeen; 45.9%) (*p*-value = 0.563). A majority of fifth-year dental students thought it most likely that abusers were parents (twenty-two; 68.75%), followed by relatives (five; 15.6%). Meanwhile, second-year dental students defined them primarily as parents (nineteen; 44.19%) and housemaids (thirteen; 30.2%); there was a statistically significant difference between fifth- and second-year students (*p*-value = 0.041) as indicated in Table 4.

## 4. Discussion

Child abuse and neglect (CAN) are worldwide problems of public health that have a substantial impact on children’s well-being and long-term health outcomes [1]. It is crucial to ensure that healthcare providers possess competent skills to address this issue. Previous research has shed light on the awareness levels among healthcare professionals in different countries [17,23,24,25,26,27,28]. A study conducted in Australia revealed that only 9.5% of participants considered themselves well-educated on the topic of CAN [23]. Additionally, a study that was conducted in Pakistan that included 575 healthcare professionals found that 78.3% agreed on the importance of documenting CAN cases, yet a significant proportion of 58.0% did not take any action when faced with suspected cases [14].

In Saudi Arabia, notable variations were observed among pediatricians in terms of CAN reporting, with percentages ranging from 43.0% to 82%, and it was noticed that those who received their education locally reported cases of CAN more frequently than those who received their education abroad [24]. Similar findings highlighted the poor knowledge among healthcare professionals regarding CAN reporting in other studies that were conducted in Saudi Arabia and other countries [17,25,26,27,28].

Teaching dental students about CAN is important, and insufficient knowledge and awareness of CAN may lead to a higher number of undetected cases [15,17,26,27,28,29]. Therefore, this study aimed to assess the awareness of CAN among dental students at King Saud University in Riyadh, Saudi Arabia, and identify their educational needs and sources of information. The findings shed light on the status of CAN awareness among dental students and highlighted the areas for improvement in their education and training.

The results of our study indicate that most dental students were aware of child abuse and neglect, with 96.0% reporting awareness. This finding is consistent with a previous study conducted on preclinical and clinical medical students, suggesting that dental students have a certain level of knowledge about CAN [22]. However, it is important to note that awareness does not necessarily translate into sufficient knowledge and skills to effectively identify and manage CAN cases. Therefore, comprehensive education and training programs are needed to enhance the student’s understanding of CAN. Moreover, most of the students in our study were reasonably aware of CAN, with the majority acknowledging its existence in Saudi Arabia. In addition, most students agreed that CAN would affect the patient’s well-being in the future, which aligns with the study findings of Al-Qahtani et al. who studied the knowledge levels and educational experiences among dental graduates in Saudi Arabia regarding child abuse and neglect [22].

Consequently, a significant number of students believed that they would have a role in dealing with CAN, and they expressed interest in handling such cases. However, nearly half of them believed that CAN is common in Saudi Arabia, contradicting the findings of the Al-Qahtani et al. study, where most students believed it to be highly prevalent and half of them were not interested in dealing with CAN cases [22].

Regarding the sources of information about child abuse and neglect, social media emerged as the most accessed source among dental students, with 85.0% reporting it as a source of information, which agrees with the Al-Qahtani et al. study, where the majority of participants believed that social media played a significant role in disseminating information about CAN [22]. Interestingly, we found that the dental college was reported as the least utilized source of information about child abuse and neglect, with only 50% of students perceiving it as a source. This finding highlights a potential gap in the dental education curriculum regarding CAN awareness and prevention. Dental schools should consider incorporating comprehensive training modules on CAN, including early recognition, reporting procedures, and interprofessional collaboration, to equip future dentists with the necessary knowledge and skills to address this issue.

Furthermore, approximately half of the participants acquired information about CAN from the dental college, which contradicts the Ivanoff et al. study which reported that classroom training in CAN caused an increased knowledge of the students [30]. However, the current study shows that the perception of the knowledge received by the participants was often described as fair, indicating a mutual agreement among the students regarding the necessity for supplementary specialized sessions and courses on CAN within the college curriculum. This is particularly crucial considering that dental students are expected to become future healthcare professionals, making the dental college an essential and reliable source of information. Several studies, both local and international, have reported comparable findings, emphasizing the importance of addressing this issue in dental education [17,22,31,32,33,34].

The study also reported that a small proportion of students, 16.0%, reported encountering cases of child abuse and neglect during their dental practice. This finding raises concerns about the recognition and reporting of CAN cases by dental professionals. It will be important to improve dental students’ ability to recognize and respond to suspected cases of CAN through practical training and case discussions which coincide with the results of Al-Qahtani et al. [22].

Additionally, most students were aware of the existence of a legal system in Saudi Arabia to deal with CAN. This is unlike what was found in other studies where a proportion of the students did not know about the legal system or who is involved in dealing with CAN [16,17,22,24].

Moreover, half of the participants thought that administrators have a role in dealing with CAN, while the majority saw legislation and law enforcement as the most effective preventative measures, which contradicts the fact that administrators have direct involvement in legal issues, which could eventually lead to a lack of knowledge. This result was also reported by Al-Qahtani et al. [22]. Additionally, there was a significant difference between male and female students regarding their perception of the dental college as a source of information about abuse. This difference suggests the need for targeted educational interventions to ensure that all students, regardless of gender, have access to the necessary information and resources related to CAN.

Furthermore, the study revealed a significant difference in awareness and knowledge between preclinical (2nd year) and clinical (5th year) dental students. Fifth-year students showed higher levels of awareness and knowledge, indicating that the dental curriculum may provide more exposure to and education on CAN in the later years which is similar to Al-Qahtani et al.’s findings [22]. However, it is important to address this gap and ensure that all dental students receive comprehensive training on CAN from the early stages of their education. In the current study, the participants recognized the importance of addressing child abuse and neglect in the dental field, with more than 90.0% of students considering it an important topic. This finding emphasizes the potential role of dentists in identifying and intervening in cases of CAN. 

The majority of students expressed interest in learning the best ways to deal with child abuse and neglect cases, indicating their willingness to enhance their knowledge and skills in this area. This presents an opportunity for dental schools to develop and implement comprehensive educational programs that cover the identification, management, and reporting of CAN cases.

The findings of this study suggest the need for improvements in the dental education curriculum regarding child abuse and neglect. Dental schools should consider incorporating comprehensive and standardized training programs on CAN, ensuring that students have the necessary knowledge, skills, and resources to identify, report, and address cases of abuse and neglect, consistent with previous research found among qualified practitioners in activity [10]. 

The limitations of this study are as follows: (1) the self-reported questionnaire, which could lead to social desirability bias, so one of the concerns is response bias, which is an individual’s susceptibility to respond in a certain way regardless of the question; (2) The relatively small sample size. Therefore, the findings should be interpreted considering these limitations, considering that they represent the sample size; (3) Additionally, the use of an online questionnaire may have introduced some potential drawbacks in terms of data quality when compared to alternative questionnaire methods. However, the online survey methodology was chosen for its convenience and accessibility to all participants, considering the limitations imposed by the study’s scope and resources; (4) Also, this research was conducted exclusively in a single dental college in Saudi Arabia, and thus, expanding this investigation to encompass all national dental colleges would be valuable, facilitating a comprehensive, country-wide study. Although child abuse and neglect educational lectures are given during the dental undergraduate years, this study shows that there is an eminent need to provide more comprehensive educational programs starting with the first years of undergraduate studies.

## 5. Conclusions

In conclusion, through this study, some students at the end of their course show an awareness of the reality of CAN at the oral level. However, not being trained to deal with different types of CAN cases, they remain powerless. To remedy this state of affairs, after having defined the needs and gaps existing at the level of the dental training curriculum, the development and better design of clinical courses are necessary.

## Figures and Tables

**Table 1:** Demographic details of the participants and awareness of CAN.

Characteristic	Value	(n)	(%)
Academic year	2nd dental year	43	57.3
	5th dental year	32	42.7
Age	19–20	26	34.7
	21–22	19	25.3
	23–24	29	38.7
	25 or more	1	1.3
Sex	Male	38	50.7
	Female	37	49.3
Marital status	Single	73	97.3
	Married	2	2.7
Nationality	Saudi	75	100.0
	Non-Saudi	0	00.0
Are you aware of child abuse and neglect?	Yes	72	96.0
	No	3	4.0
Source of information on child abuse and neglect (Multiple responses allowed)	Social media	64	85.3
	Dental College	38	50.7
	Relatives and friends	40	53.3
	Mass media	45	60.0
Have you taken an academic course(s) or session(s) about child abuse and neglect in your Dental college?	Yes	37	49.3
	No	38	50.7

**Table 2:** Participant’s opinions on the prevalence of CAN in Saudi population and their responsibility in the issue.

Characteristic	Value	(n)	(%)
Do you think the topic of child abuse and neglect is important in the dental field?	Yes	70	93.3
	No	2	2.7
	Uncertain	3	4.0
Have you seen or encountered a case of child abuse and neglect during your dental study?	Yes	18	24.0
	No	57	76.0
Do you believe child abuse and neglect exist in Saudi’s population?	Yes	71	94.7
	No	0	0.0
	Uncertain	4	5.3
How common do you think child abuse and neglect is in Saudi’s population?	Very common	7	9.3
	Common	26	34.7
	Not common (neutral)	31	41.3
	Rare	11	14.7
Do you think child abuse and neglect will affect his/her future?	Yes	72	96.0
	No	2	2.7
	I don’t know	1	1.3
As a future dentist, generally, in any discipline, do you think you have a role in child abuse and neglect?	Yes	59	78.7
	No	3	4.0
	I don’t know	13	17.3
Do you have an interest to know the best way when dealing with child abuse and neglect?	Yes	66	88.0
	No	1	1.3
	Uncertain	8	10.7
How good do you think those academic course(s) or session(s) about child abuse and neglect were?	Excellent	7	9.3
	Good	11	14.7
	Fair	37	49.3
	Sufficient	9	12.0
	Insufficient	11	14.7
Do you like to receive more courses and sessions about child abuse and neglect?	Yes	53	70.7
	No	9	12.0
	Uncertain	13	17.3

**Table 3:** Participants’ responses on the legal ways CAN is dealt and preventive measures.

Characteristic	Value	(n)	(%)
Who do you think deals with child abuse and neglect? (Multiple responses allowed)	Social workers	60	80.0
	Physicians	60	80.0
	Police	57	76.0
	Nurses	44	58.7
	Administrators	35	46.7
Do you think there is a legal system–legislation—to deal with child abuse and neglect in the Kingdom of Saudi Arabia?	Yes	71	94.7
	No	4	5.3
Who do you think abuse children the most (the abuser)?	Parents	3	4.0
	Relatives (excluding parents)	14	18.7
	Housemaids	41	54.7
	Drivers	12	16.0
	Teacher	5	6.7
Do you think that the child abuser has a psychological disorder?	Always	17	22.7
	Commonly	31	41.3
	Sometimes	13	17.3
	Rarely	7	9.3
	Never	1	1.3
	I don’t know	6	8.0
What do you think are the most effective ways to increase awareness about child abuse and neglect?	Dental staff education	6	8.0
	Legalization and law enforcement	35	46.7
	Public education	28	37.3
	School education	6	8.0
If detect a case of child abuse in your clinic, what is your first plan of action?	Ignore	1	1.3
	Parent counseling	31	41.3
	Refer to police as a medico-legal case	33	44.0
	Referral	5	6.7
	Treatment of presenting symptoms	5	6.7

**Table 4:** Comparison between genders and academic years with each characteristic.

Characteristic	Value	Gender		*p*-value	Academic Year		*p*-Value
		Males n (%) 38 (50.7%)	Female n (%) 37 (49.3%)		2nd Year n (%) 43 (57.3%)	5th Year n (%) 32 (42.7%)	
As a future physician, generally, at any discipline, do you think you have a role in child abuse and neglect?	Yes	30 (78.9%)	29 (78.4%)	0.813	30 (69.8%)	29 (90.6%)	0.071
	No	1 (2.6%)	2 (5.4%)		3 (7.0%)	0 (0.0%)	
	I don’t know	7 (18.42%)	6 (16.22%)		10 (23.2%)	3 (9.38%)	
Do you have an interest to know the best way when dealing with child abuse and neglect?	Yes	34 (89.5%)	32 (86.5%)		37 (86.0%)	29 (90.6%)	
	No	1 (2.6%)	0 (0.0%)	0.461	1(2.3%)	0 (0.0%)	0.646
	Uncertain	3 (7.89%)	5 (13.51%)		5 (11.63%)	3 (9.38%)	
Have you taken an academic course(s) or session(s) about child abuse and neglect in your Dental college?	Yes	20 (52.6%)	17 (45.9%)	0.563	6 (14.0%)	31 (96.9%)	0.000 *
	No	18 (47.4%)	20 (54.1%)		37 (86.0%)	1 (3.1%)	
How good do you think those academic course(s) or session(s) about child abuse and neglect were?	Excellent	1 (2.6%)	6 (16.2%)	0.147	3 (7.0%)	4 (12.5%)	0.182
	Good	7 (18.4%)	4 (10.8%)		4 (9.3%)	7 (21.9%)	
	Fair	22(57.89)	15 (40.54%)		23 (53.4%)	14 (43.75%)	
	Sufficient	3 (7.89%)	6 (16.2%)		4 (9.30%)	5 (15.6%)	
	Insufficient	5 (13.16%)	6 (16.2%)		9 (20.93%)	2 (6.3%)	
Do you think there is a legal system—legislation—to deal with child abuse and neglect in the Kingdom of Saudi Arabia?	Yes	37 (97.4%)	34 (91.9%)	0.291	39 (90.7%)	32 (100.0%)	0.076
	No	1 (2.6%)	3 (8.1%)		4 (9.3%)	0 (0.0%)	
Who do you think deals with child abuse and neglect? (Multiple responses allowed)	Social workers	26 (68.4%)	34 (91.9%)	0.011 *	33 (76.7%)	27 (84.4%)	0.414
	Physicians	32 (84.2%)	28 (75.7%)	0.356	33 (76.7%)	27 (84.4%)	0.414
	Police	31 (81.6%)	26 (70.3%)	0.252	34 (79.1%)	23 (71.9%)	0.471
	Nurses	25 (65.8%)	19 (51.4%)	0.204	26 (60.5%)	18 (56.3%)	0.714
	Administrators	18 (47.4%)	17 (45.9%)	0.902	23 (53.5%)	12 (37.5%)	0.170
Who do you think abuse children the most (the abuser)?	Drivers	1 (2.6%)	2 (5.4%)	0.884	1 (2.3%)	2 (6.3%)	0.041 *
	Housemaids	6 (15.8%)	8 (21.6%)		13 (30.2%)	1 (3.1%)	
	Parents	21 (55.26%)	20 (54.05%)		19 (44.19%)	22 (68.75%)	
	Relatives	7 (18.42%)	5 (13.5%)		7 (16.28%)	5 (15.6%)	
	Teacher	3 (7.89%)	2 (5.4%)		3 (6.98%)	2 (6.3%)	
Do you think that the child abuser has a psychological disorder?	Always	6 (15.8%)	11 (29.7%)		9 (20.9%)	8 (25.0%)	
	Commonly	13 (34.2%)	18 (48.6%)		18 (41.9%)	13 (40.6%)	
	Sometimes	8 (21.05%)	5 (13.51%)	0.146	8 (18.60%)	5 (15.63%)	0.71
	Rarely	6 (15.79%)	1 (2.7%)		5 (11.63%)	2 (6.3%)	
	Never	1 (2.63%)	0 (0.0%)		1 (2.33%)	0 (0.0%)	
	I don’t know	4 (10.5%)	2 (5.4%)		2 (4.7%)	4 (12.5%)	
What do you think are the most effective ways to increase awareness about child abuse and neglect?	4 (10.5%)	2 (5.4%)	4 (10.5%)	0.718	3 (7.0%)	3 (9.4%)	0.438
	18 (47.4%)	17 (45.9%)	18 (47.4%)		23 (53.5%)	12 (37.5%)	
	14 (36.84%)	14 (37.8%)	14 (36.84%)		13 (30.23%)	15 (46.88%)	
	2 (5.26%)	4 (10.8%)	2 (5.26%)		4 (9.30%)	2 (6.3%)	
If detected a case of child abuse in your clinic, what is your first plan of action?	Ignore	5 (0.0%)	1 (2.7%)		1 (2.3%)	0 (0.0%)	
	Parent counseling	18 (47.4%)	13 (35.1%)	0.225	18 (41.9%)	13 (40.6%)	0.848
	Refer to police as the medico-legal case	15 (39.47)	18 (48.65%)		19 (44.19%)	14 (43.75%)	
	Referral	1 (2.63%)	4 (10.8%		3 (6.98%)	2 (6.3%)	
	Treatment of presenting symptoms	4 (10.53%)	1 (2.7%)		2 (4.65%)	3 (9.4%)	

* (*p* < 0.05). Statistics: Chi-square analysis.

## Data Availability

Data supporting the findings of the present study can be requested from the authors. The data are not publicly available.

## References

[1] Petersen A.C., Joseph J., Feit M., Institute of Medicine & National Research Council (2014). Committee on Child Maltreatment Research, Policy, and Practice for the Next Decade: Phase II, Board on Children, Youth, and Families, Committee on Law and Justice. New Directions in Child Abuse and Neglect Research.

[2] Assink M., van der Put C.E., Meeuwsen M.W.C.M., de Jong N.M., Oort F.J., Stams G.J.J.M., Hoeve M. (2019). Risk factors for child sexual abuse victimization: A meta-analytic review. Psychol. Bull..

[3] World Health Organization (2022). Child Maltreatment. https://www.who.int/news-room/fact-sheets/detail/child-maltreatment.

[4] Sharratt K., Mason S.J., Kirkman G., Willmott D., McDermott D., Timmins S., Wager N.M. (2023). Childhood Abuse and Neglect, Exposure to Domestic Violence and Sibling Violence: Profiles and Associations with Sociodemographic Variables and Mental Health Indicators. J. Interpers. Violence.

[5] U.S. Department of Health & Human Services, Administration for Children and Families, Administration on Children, Youth and Families, Children’s Bureau (2023). Child Maltreatment 2021. https://www.acf.hhs.gov/cb/data-research/child-maltreatment.pdf.

[6] Stoltenborgh M., Bakermans-Kranenburg M.J., Alink L.R.A., van Ijzendoorn M.H. (2014). The Prevalence of Child Maltreatment across the Globe: Review of a Series of Meta-Analyses. Child Abus. Rev..

[7] Al-Eissa M.A., AlBuhairan F.S., Qayad M., Saleheen H., Runyan D., Almuneef M. (2014). Determining child maltreatment incidence in Saudi Arabia using the ICAST-CH: A pilot study. Child Abus. Negl..

[8] Nilchian F., Tarrahi M.J., Zare N. (2021). A systematic review and meta-analysis of failure to take history as a barrier of reporting child abuse by dentists in private and state clinics. Dent. Res. J..

[9] UNICEF (2009). Child Abuse: A Painful Reality Behind Closed Doors. Challenges: Newsletter on Progress towards the Millenium Development Goals from a Child Rights Perspective.

[10] Mogaddam M., Kamal I., Merdad L., Alamoudi N. (2016). Knowledge, attitudes, and behaviors of dentists regarding child physical abuse in Jeddah, Saudi Arabia. Child Abus. Negl..

[11] Nagarajan S.K. (2018). Craniofacial and oral manifestation of child abuse: A dental surgeon’s guide. J. Forensic Dent. Sci..

[12] Buldur B., Büyükkök Ç., Cavalcanti A.L. (2022). Knowledge, attitudes, and perceptions regarding child abuse and neglect among dentists in Turkey. Braz. Oral. Res..

[13] Sulimany A.M., Alsamhan A., Alawwad A.A., Aqueel M., Alzaid N., Bawazir O.A., Hamdan H.M. (2021). Knowledge Levels and Educational Experiences among Dental Graduates in Saudi Arabia Regarding Child Abuse and Neglect: A National Study. Children.

[14] Ali Khan H.M., Mansoori N., Sohail M.H., Humayun M.A., Liaquat A., Mubeen S.M. (2021). Child physical abuse: Awareness and practices of medical and dental doctors in Pakistan. JPMA. J. Pak. Med. Assoc..

[15] Orhon F.S., Ulukol B., Bingoler B., Gulnar S.B. (2006). Attitudes of Turkish parents, pediatric residents, and medical students toward child disciplinary practices. Child Abus. Negl..

[16] Domestic Violence and Child Abuse and Neglect in Saudi Arabia. https://nfsp.org.sa/ar/excellencecentre/Pages/publications.aspx.

[17] Alanazi S.S., Althaqib A.N., Albeladi K.E., Alarfaj S., Alhezemy R.R., Almjlad A.B., Farhan M.A., Albuhairi S.I. (2021). Child abuse and neglect awareness between knowledge, perception, and reporting among interns and medical students of Majmaah University. IJMDC.

[18] Ortiz-Tallo M., Calvo I. (2020). Child sexual abuse: Listening to the victims. Arch. Community Med. Public. Health.

[19] John V., Messer L.B., Arora R., Fung S., Hatzis E., Nguyen T., San A., Thomas K. (1999). Child abuse and dentistry: A study of knowledge and attitudes among dentists in Victoria, Australia. Aust. Dent. J..

[20] Cairns A.M., Mok J.Y., Welbury R.R. (2005). Injuries to the head, face, mouth and neck in physically abused children in a community setting. Int. J. Paediatr. Dent..

[21] Fowler F.J. (2009). Survey Research Methods. Applied Social Research Methods.

[22] Al-Qahtani M.H., Almanamin H.H., Alasiri A.M., Alqudaihi M.H., AlSaffar M.H., Yousef A.A., Awary B.H., Albuali W.H. (2022). Child Abuse and Neglect Awareness among Medical Students. Children.

[23] Kraus C., Jandl-Jager E. (2011). Awareness and knowledge of child abuse amongst physicians–A descriptive study by a sample of rural Austria. Wien. Klin. Wochenschr..

[24] Alnasser Y., Albijadi A., Abdullah W., Aldabeeb D., Alomair A., Alsaddiqi S., Alsalloum Y. (2017). Child Maltreatment between Knowledge, Attitude and Beliefs among Saudi Pediatricians, Pediatric Residency Trainees and Medical Students. Ann. Med. Surg..

[25] Alsaleem S., Alsaleem M., Asiri A.M., Alkhidhran S.S., Alqahtani W.S.S., Alzahrani M.S., Assiri H., Alshahrany K.M., Alsamghan A.S. (2019). Knowledge and Attitude Regarding Child Abuse among Primary Health Care Physician in Abha, Saudi Arabia, 2018. J. Family Med. Prim. Care.

[26] Aldukhayel A., Aljarbou E., Alturki F.M., Almazyad N.S., Alsaqer O.M., Almutairi R., Aldukhayel A., Aljarbooa E., Alturki F.M., Almazyad N.S. (2020). Knowledge and Attitude Regarding Child Abuse Among Primary Healthcare Physicians and Interns in Al Qassim, Saudi Arabia. Cureus.

[27] Gopalakrishna V., Basheer B., Alzomaili A., Aldaham A., Abalhassan G., Almuziri H., Alatyan M., AlJofan M., Al-Kaoud R. (2020). Knowledge and Attitudes toward Child Abuse and Neglect among Medical and Dental Undergraduate Students and Interns in Riyadh, Saudi Arabia. Imam J. Appl. Sci..

[28] Nihan K., Makda A., Salat H., Khursheed M., Fayyaz J., Khan U. (2021). Assessment of Knowledge, Attitude, and Practice of Child Abuse amongst Health Care Professionals Working in Tertiary Care Hospitals of Karachi, Pakistan. J. Family Med. Prim. Care.

[29] Saeed N., Anwar E., Salama N., Galal M., Ghanem M. (2021). Child Maltreatment: Knowledge, Attitudes and Reporting Behaviour of Physicians in Teaching Hospitals, Egypt. East. Mediterr. Health J..

[30] Ivanoff C.S., Andonov B., Hottel T.L. (2023). Dental Students’ Knowledge and Reporting of Child Maltreatment: Where Are We at Today Both Here and Abroad?. Eur. J. Dent. Educ..

[31] Soldatou A., Stathi A., Panos A., Paouri B., Koutsoukou E., Krepis P., Tsolia M., Oral R., Leventhal J.M. (2020). A National Educational Campaign to Raise Awareness of Child Physical Abuse among Health Care Professionals. Eur. J. Pediatr..

[32] Li X., Yue Q., Wang S., Wang H., Jiang J., Gong L., Liu W., Huang X., Xu T. (2017). Knowledge, Attitudes, and Behaviours of Healthcare Professionals Regarding Child Maltreatment in China. Child. Care Health Dev..

[33] Kara Ö., Çalişkan D., Suskan E. (2014). Comparison of the Levels of Knowledge and Approaches in Relation with Child Abuse and Neglect in Residents of Pediatrics, Pediatricians and Practitioners Working in the Province of Ankara. Turk. Pediatri Ars..

[34] Flaherty E.G., Schwartz K., Jones R.D., Sege R.D. (2013). Child Abuse Physicians: Coping with Challenges. Eval. Health Prof..

